# Intracystic papillary carcinoma associated with ductal carcinoma in situ in a male breast: a case report

**DOI:** 10.4076/1757-1626-2-7260

**Published:** 2009-07-29

**Authors:** Sami Aziz Brahmi, Fatema Zahra El M’rabet, Yusra Akesbi, Zineb Benbrahim, fatemi El Hind, Kawtar Znati, Amal Benlemlih, Naima Tbaili, Mustapha Maaroufi, Siham Tizniti, Afaf Amarti, Omar El Mesbahi

**Affiliations:** 1 Medical Oncology unit, Hassan II University HospitalFezMorocco; 2 Department of Anatomopathology, Hassan II University HospitalFezMorocco; 3 Department of Radiology, Hassan II University HospitalFezMorocco; 4 Department of Gynecology, Ibn Khatib HospitalFezMorocco

## Abstract

**Introduction:**

Intracystic papillary carcinoma represents a small distinctive subgroup of noninvasive breast cancer, accounts for <0.5% of breast malignancies and is extremely rare in men, it was originally reported as a localized non-invasive carcinoma, but is usually associated with ductal carcinoma in situ around the main tumor or invasive carcinoma.

**Case presentation:**

We report a case of 50-year-old man with intracystic papillary carcinoma in man with ductal carcinoma in situ who underwent a tumorectomy following by a radical Patey intervention (Halsted).

**Conclusion:**

Nowadays, there is still no clear consensus regarding optimal treatment of intracystic papillary carcinoma. Most papers reinforce the importance of an adequate surgical margin in conservative treatment. Surgeons must pay much attention to the potential for ductal carcinoma in situ around the tumor when selecting the operative procedure.

## Introduction

Male breast cancer is a rare disease and the incidence is 1% of all breast cancer [1]. Intracystic papillary carcinoma (IPC) represents a small distinctive subgroup of noninvasive breast cancer, accounts for <0.5% of breast malignancies and is extremely rare in men [1]. It was originally reported as a localized non-invasive carcinoma, but is usually associated with ductal carcinoma in situ (DCIS) around the main tumor or invasive carcinoma [1]. We report a case of 50-year-old man with IPC in man with DCIS.

## Case presentation

**Figure 1.**
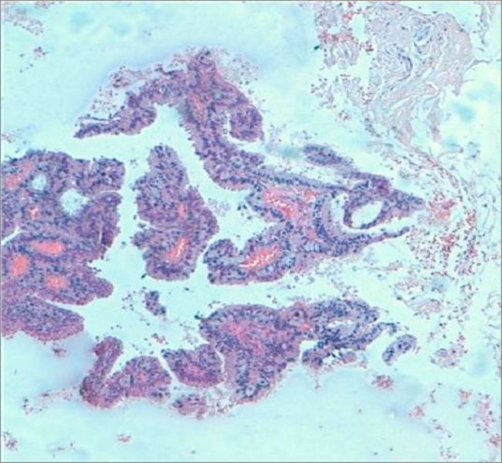
Low-power photomicrograph of a histopathologic specimen showing fibrotic cyst wall and papillary proliferation.

**Figure 2.**
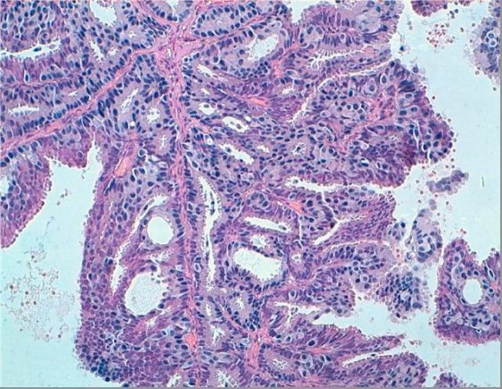
High-power photomicrograph showing a papillary frond with a fibrovascular core.

**Figure 3.**
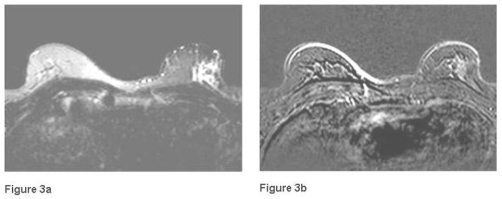
Axial T2-weighted breast MRI **(a)** et T1-weighted MRI with injection of gadolinium **(b)** showing a retromamelonnar speculated left lesion measuring 24 mm with a central marked enhancement.

In this report, we present a rare case of a Moroccan, 50-year-old Arabic man. He presented with a 2-month history of a small mass located in the left retroareolar region of the breast and visited a community hospital where he underwent a tumorectomy. Histopathological examination revealed a cyst containing a papillary proliferation. Low-power photomicrograph of a histopathologic specimen showed fibrotic cyst wall and papillary proliferation (Figure 1), high-power photomicrograph showed a papillary frond with a fibrovascular core, the epithelial cells are pleomorphic (Figure 2) and the final diagnosis was intracystic papillary carcinoma, intermediate grade. Both estrogen receptor and progesterone receptor were positive. By the end, the patient was referred to Hassan II University Hospital for additional treatment. In our center a radiological assessment was done. Mammogram was normal and ultrasound revealed a heterogenous speculated left lesion in the junction of the inner quadrants with left axilar adenopathies. Magnetic resonance imagery showed a retromamelonnar speculated left lesion measuring 24 mm (Figure 3a) with a central marked enhancement (Figure 3b). This lesion had a contact with pectoral muscle (Figure 3b). For this reason, we decided to perform a radical mastectomy (Halsted) with axillary lymph node dissection. On histopathological examination, a focus of ductal carcinoma in situ was shown. There were no metastases in the right axillary lymph nodes. Postoperatively, no further treatment was performed, and the patient has remained well for 7 months without any signs of tumor recurrence.

## Discussion

Intracystic breast carcinoma is rare in females and exceedingly rare in males with a handful of case reports in the literature. Approximately half of IPCs arise in the retroareolar region of the breast and the usual clinical manifestation is a palpable mass or nipple discharge [2]. It commonly presents as a benign-appearing due to its underlying cystic nature [3]. In a case report and subsequent review of Japanese cases in men, Tochika et al [4] reported the mean age of intracystic carcinoma in males as 68.2 years and most of the patients presented with a palpable lump.

Radiological studies are helpful. IPC tends to be well defined on mammography; an irregular margin suggests the presence of invasion [5]. Ultrasonography typically reveals a hypo-echoic area (representing the cyst) with soft tissue echoes projecting from wall of the cyst (intracystic tumour) [6]. Contrast-enhanced MRI may show marked enhancement of cyst walls, septations, and mural nodules [7].

Intracystic papillary carcinoma can occur in a pure form, or it may be associated with ductal carcinoma in situ or invasive carcinoma not otherwise specified [8]. Pathologically, intracystic papillary carcinomas may show four cellular patterns: cribriform, compact columnar epithelial, stratified spindle cell or a transitional cell form resembling urothelium, or a combination of two or more of these patterns may be seen [9]. The majority of patients with IPC will have associated DCIS or invasive cancer or both, and should be treated on the basis of this associated pathology [10]. Some studies have suggested that core needle biopsy (CNB) has been proved to be more effective in distinguishing papillary neoplasms from other diseases and benign papillomas from papillary carcinoma [10]. The differential diagnosis of intracystic papillary lesions should be made on histopathological specimens. Tsuda et al [11] reported that loss of heterozygosity (LOH) on chromosome 16q was a useful marker for intracystic papillary carcinoma, since intraductal papilloma showed no LOH. Using this method by the polymerase chain reaction, the malignant potential of intracystic papillary lesions may be more clearly determined.

The nature of the associated lesions to IPC is essential for prognostic reasons and for assessment of the margins. Moreover, IPC accompanied by DCIS is an important precursor to invasive carcinoma and further treatment is indicated, if medically feasible [12].

Nowadays, there is still no clear consensus regarding optimal treatment of IPC. Most papers reinforce the importance of an adequate surgical margin in conservative treatment. It is speculated that nearly half of the IPC cases may be treated by breast conservative surgery, although surgeons must pay much attention to the potential for DCIS around the tumor when selecting the operative procedure [12]. It is true that low frequency of axillary node metastases with pure IPC does not justify axillary lymph node dissection. Although, the role of sentinel node biopsy has not been evaluated in this disease, it seems that sentinel node biopsy may be an excellent alternative to full axillary dissection in patients with IPC and associated DCIS or invasive carcinoma [12].

In the present case, initially tumorectomy was performed. However, IPC was associated with DCIS around the tumor detected by radiologic test. It was necessary to perform radical mastectomy and axillary lymph node dissection. We did not consider that adjuvant treatment was necessary in the presence of adequate local control and in the absence of metastatic spread of disease.

## Conclusion

There are no guidelines for management of IPC. Most papers reinforce the importance of an adequate surgical margin in conservative treatment. Surgeons must pay much attention to the potential for DCIS around the tumor when selecting the operative procedure.

## Abbreviations

CNB: core needle biopsy
DCIS: ductal carcinoma in situ
IPC: intracystic papillary carcinoma
LOH: loss of heterozygosity
MRI: magnetic resonance imaging
